# Fluoride Induces Endoplasmic Reticulum Stress in Ameloblasts Responsible for Dental Enamel Formation

**DOI:** 10.1074/jbc.M503288200

**Published:** 2005-04-23

**Authors:** Kaori Kubota, Daniel H. Lee, Masahiro Tsuchiya, Conan S. Young, Eric T. Everett, Esperanza A. Martinez-Mier, Malcolm L. Snead, Linh Nguyen, Fumihiko Urano, John D. Bartlett

**Affiliations:** ‡ Department of Cytokine Biology, Forsyth Institute,; § Department of Oral and Developmental Biology, Harvard School of Dental Medicine, Boston, Massachusetts 02115,; ¶ Division of Aging and Geriatric Dentistry, Tohoku University Graduate School of Dentistry, Sendai, 980-8575, Japan,; ‖ Department of Pediatric Dentistry and the Carolina Center for Genome Sciences, University of North Carolina, North Chapel Hill, North Carolina 27599,; ** Department of Preventive and Community Dentistry, Oral Health Research Institute, Indiana University School of Dentistry and Medicine, Indianapolis, Indiana 46202,; ‡‡ Center for Craniofacial Molecular Biology, University of Southern California School of Dentistry, Los Angeles, California 90033,; §§ Program in Gene Function and Expression, Program in Molecular Medicine, University of Massachusetts Medical School, Worcester, Massachusetts 01655

## Abstract

The mechanism of how fluoride causes fluorosis remains unknown. Exposure to fluoride can inhibit protein synthesis, and this may also occur by agents that cause endoplasmic reticulum (ER) stress. When translated proteins fail to fold properly or become misfolded, ER stress response genes are induced that together comprise the unfolded protein response. Because ameloblasts are responsible for dental enamel formation, we used an ameloblast-derived cell line (LS8) to characterize specific responses to fluoride treatment. LS8 cells were growth-inhibited by as little as 1.9–3.8 ppm fluoride, whereas higher doses induced ER stress and caspase-mediated DNA fragmentation. Growth arrest and DNA damage-inducible proteins (GADD153/CHOP, GADD45*α*), binding protein (BiP/glucose-responsive protein 78 (GRP78), the non-secreted form of carbonic anhydrase VI (CA-VI), and active X-box-binding protein-1 (Xbp-1) were all induced significantly after exposure to 38 ppm fluoride. Unexpectedly, DNA fragmentation increased when GADD153 expression was inhibited by short interfering RNA treatment but remained unaffected by transient GADD153 overexpression. Analysis of control and GADD153^−/−^ embryonic fibroblasts demonstrated that caspase-3 mediated the increased DNA fragmentation observed in the GADD153 null cells. We also demonstrate that mouse incisor ameloblasts are sensitive to the toxic effects of high dose fluoride in drinking water. Activated Ire1 initiates an ER stress response pathway, and mouse ameloblasts were shown to express activated Ire1. Ire1 levels appeared induced by fluoride treatment, indicating that ER stress may play a role in dental fluorosis. Low dose fluoride, such as that present in fluoridated drinking water, did not induce ER stress.

Fluoride is an effective caries prophylactic. However, acute or chronic exposure to fluoride can result in enamel (1) and skeletal fluorosis (2), renal toxicity (3), and epithelial lung cell toxicity (4). Fluoride is present in fresh water at concentrations of less than 0.1 ppm to >100 ppm, and concentrations of ~1.6–1.8 ppm in drinking water are the threshold for fluorosis risk among the population (5). Fluoride ingestion between the ages of 15 and 30 months may be the most critical for fluorosis of the esthetically important human maxillary central incisors (6, 7). It is during this time that dental enamel forms on the unerupted permanent teeth.

Rodents, including mice and rats, have continuously erupting incisors that manifest each developmental stage of enamel formation (amelogenesis). Moving from the distal tip of the incisor back to where the incisor grows beneath the molars, the developmental stages become progressively less mature. The initial stage of enamel development is the secretory stage. The columnar ameloblast cells of the enamel organ are responsible for dental enamel development. During the secretory stage the ameloblasts are tall, contain an extensive endoplasmic reticulum (ER),^1^ and secrete large amounts of protein into the enamel matrix. During the maturation stage the ameloblasts are short, and in contrast to the secretory stage, they absorb proteins from the enamel matrix (8). In 1977 Smith and Warshawsky (9) demonstrated that during the transition between the secretory and maturation stages (transition stage) an approximate 25% loss of rat incisor ameloblasts occurs with another 25% loss before the completion of the maturation stage. Subsequent studies confirmed the presence of apoptotic ameloblasts associated with normal rodent incisor development (10–13).

Although enamel fluorosis research has spanned seven decades (14), no consensus exists as to the mechanism of its cause. One school of thought presents compelling evidence that fluorosis is caused primarily by the presence of excessive fluoride within the forming enamel. The fluoride ions are postulated to adversely affect the precipitation of hydroxyapatite that forms the enamel structure (15). Although this postulate has merit, it does not adequately explain why different inbred strains of mice had different susceptibilities/resistance to enamel fluorosis, whereas the overall levels of fluoride present in their erupted incisors did not differ significantly (16). Additionally, fluoride concentrations in enamel from unerupted human molars did not correlate positively with fluorosis severity, and it was concluded that individual genetic variation likely plays a role in fluorosis susceptibility (17).

We examined the possibility that fluoride can cause an endoplasmic reticulum (ER) stress response. ER stress occurs when nascent proteins are not folded properly and/or are misfolded, leading to the initiation of the unfolded protein response (UPR). As the unfolded proteins accumulate in the ER, the chaperone-binding protein BiP is released into the lumen and binds to hydrophobic regions on the surface of the unfolded proteins to facilitate proper folding (18). The UPR can activate three different primary ER stress response pathways (18–20). The only pathway that is conserved among all eukaryotic cells is that initiated by Ire1 (21). Ire1 is both a kinase and an endoribonuclease. ER stress initiates Ire1 autophosphorylation and subsequent RNase activity specific for spliceosome-independent processing of Xbp1 mRNA. Processing of the Xbp1 mRNA by Ire1 results in a translation frameshift that allows encoding of active Xbp1 (22, 23). Active Xbp1 is a basic leucine zipper (bZIP) transcription factor that can bind to and initiate transcription from both the ER stress response element (ERSE) and the UPR element (23, 24). The UPR will also adapt to ER stress by translation attenuation, by protein degradation, or finally, by apoptosis. Although GADD45*α* is not known to be part of the UPR, it is a cell cycle checkpoint protein that arrests cells at G_2_/M phase (25). GADD153/CHOP is an ER stress response gene that can also be induced by other stressors such as amino acid deprivation and exposure to oxidants (26, 27). The GADD153*/*CHOP gene encodes a bZIP C/EBP homologous protein that forms heterodimers with other CCAAT enhancerbinding proteins (C/EBP) (28). When phosphorylated by p38 mitogen-activated protein kinase, GADD153 becomes a more potent inducer of apoptosis. GADD34 was recently demonstrated to be directly activated by GADD153 (29). Even so, the downstream effects of GADD153 expression are not well characterized (20).

In this study we utilize the LS8 ameloblast cell line that was derived from mouse enamel organ (30) to assess whether fluoride can induce ER stress and initiate the UPR. We present evidence that fluoride induces an ER stress response involving caspases and increased expression levels of Bip, Xbp-1, GADD153, GADD45*α*, Ire1, and the non-secreted form of carbonic anhydrase VI (CA-VI).

## EXPERIMENTAL PROCEDURES

### Cell Culture—

The mouse ameloblast-derived cell line (LS8) and the CHOP^−/−^ and CHOP^+/+^ (referred to as GADD153^−/−^ or -^+/+^) mouse embryo fibroblasts were maintained in *α* minimal essential medium (Invitrogen) supplemented with fetal bovine serum (10%), penicillin (50 units/ml), and streptomycin (50 *μ*g/ml).

### Sodium Fluoride Treatment and Determination of Cell Growth and Viability—

To assess cell viability and growth, survival and 3-(4,5-dimethylthiazol-2-yl)-2,5-diphenyltetrazolium bromide (MTT) assays were performed. For survival assays, LS8 cells were plated at a density of 1000 cells in T-25 cm^2^ flasks for 18 h and exposed to concentrations of NaF (stock, 100× in water) from between 0 and 2 mm for a period of 24 h. Cells were washed with phosphate-buffered saline and allowed to grow in fresh medium for ~8–9 days. The resulting colonies were stained with 0.5% methylene blue in 50% methanol and counted. Percent cell survival was then calculated (31, 32).

For MTT assays, cells were plated in 96-well plates, and the experiments were performed after 18 h. The indicated concentrations of NaF were added to the wells. After 24 and 48 h, cell growth was determined by measuring MTT (Sigma) reductase activity. Briefly, MTT (0.5 mg/ml final concentration) was added and incubated for 3.5 h. After removal of the medium, crystals were dissolved in dimethyl sulfoxide (Me_2_SO) (Sigma), and optical density was measured at 550 nm using a microplate reader (HTS 7000 Bio Assay Reader, PerkinElmer Life Sciences). Six wells were analyzed, and the mean value was calculated for each NaF concentration. Experiments were performed in triplicate.

### DNA Fragmentation ELISA Assay—

DNA fragmentation was assayed using the Cell Death Detection ELISA kit (Roche Diagnostics). Cells were incubated in the presence or absence of either 50 *μ*m z-VAD-fluoromethyl ketone (Promega) or 2 *μ*m z-DEVD-fluoromethyl ketone (BioVision) for 1 h at 37 °C and then treated with NaF for 24 or 48 h followed by processing for the Cell Death ELISA assay. The procedure was performed according to the manufacturer’s instructions. All assays were performed in triplicate.

### Reverse Transcriptase-Polymerase Chain Reaction—

Total RNA was extracted from LS8 cells with Trizol reagent (Invitrogen), and cDNA was prepared using the SuperScript first-strand synthesis system (Invitrogen). PCR primers were: CA-VI Type A (sense), 5′-AGTGCTGGGCTTAGTTTAGAGCTTTCC-3′, CA-VI Type B (sense), 5′-TCCTGCATTCAGGGCTACAGCATCTG-3′, and CA-VI type A and B (antisense), 5′-AGATCGATCGATACTGTGTGTCCGT-3′.

### Northern Blot Analysis—

The GADD153, BiP, GADD45*α*, XBP-1, *β*-actin, and CA-VI type B cDNA fragments were labeled with [*α*-^32^P]dCTP (6000 Ci/mmol) (PerkinElmer Life Sciences) using Prime-It RmT random primer labeling kit (Stratagene). In brief, 50 ng of PCR product was added to the reagent mix (containing random primers and dNTPs) and boiled for 5 min. Ten *μ*l of [*α*-^32^P]dCTP and 3 *μ*l of magenta DNA polymerase were added to the boiled mix and incubated at 37 °C for 10 min. The labeled cDNA fragment was denatured and added to the hybridization solution. Ten *μ*g of total RNA was run on a formaldehyde-agarose gel and transferred to a Hybond-N nylon membrane (Amersham Biosciences). The membrane was prehybridized in hybridization solution with 0.1 mg/ml heat-denatured salmon sperm DNA (Invitrogen) at 65 °C for 1 h. ^32^P-Labeled cDNA was added, and the membrane was incubated at 65 °C overnight. The membrane was washed (0.1 × SSC (1× SSC = 0.15 m NaCl and 0.015 m sodium citrate), 0.5% SDS) at 65 °C 3 times for 30 min each. Membranes were stripped and re-probed for the indicated mRNAs.

### Extraction and Treatment of Primary Porcine Enamel Organ Epithelial Cells—

Third molar tooth buds were removed from the mandible of a 6-month-old pig and placed in Hanks’ balanced salt solution supplemented with 0.02% EDTA for 30 min. Enamel organs were dissected from mineralizing tooth cusps and pulp organs. The enamel organs were dissociated into a single cell suspension as previously described (33) with the modification that any undissociated tissue pieces were eliminated by passing the suspension through a 40-*μ*m cell sieve (BD Biosciences). Approximately 3 × 10^6^ cells were obtained per enamel organ. Enamel organ cells were grown in Primaria T-75 flasks in LHC-8 medium (BIOSOURCE) supplemented with 10% fetal bovine serum, penicillin/streptomycin/amphotericin B, and 0.5 *μ*g/ml epinephrine at 37 °C in 5% CO_2_. After 10–12 days in culture, a fast-growing fibroblast-like cell population grew to confluency and then expired. Slow-growing epithelial cells remained. These cells were polygonal and grew as tightly clustered colonies. The cells were 80–90% confluent after an additional 20 days in culture. Cells were harvested (trypsin) from flasks, counted, and inoculated into 6-well plates at a density of 2.5 × 10^5^ cells per well. The next day duplicate wells were treated or not with 2 mm NaF for 48 h followed by collection of total RNA with Trizol^®^ reagent (Invitrogen). This procedure was performed on EO cells from two different 6-month-old pigs.

### Real-time PCR Analysis of GADD153 and BiP Expression in Primary Porcine Enamel Organ Epithelial Cells—

SuperScript III first-strand synthesis system for reverse transcription-PCR (Invitrogen) generated the cDNA for real-time PCR analysis. Primer sequence for expression analyses were: porcine BiP, forward, 5′-AAGACAAAGGTACAGGCAACAAAA-3′, reverse, 5′-CTCAGCAAACTTCTCAGCATCATT-3′; porcine GADD153, forward, 5′-CCTTGGGCTACTGCTGAC-3′, reverse, 5′-CATGAATAGAGGGGGTTGAG-3′. Internal control primers for porcine eEF1*α*1 were: forward, 5′-GATGGAAAGTCACCCGTAAAGATG-3′, and reverse, 5′-GTTGGACGAGTTGGTGGTAGAATG-3′. The PCR temperature profile was 3 min at 95 °C (initial melt) then 20 s at 95 °C, 30 s at 65 °C for 45 cycles, and 30 s at 95 °C for 1 cycle, and 1 min at 55 °C followed by stepwise temperature increases from 55 to 95 °C to generate the melt curve. Standard curves were generated with each primer set by use of untreated control cDNA preparations and a 10-fold dilution series ranging from 1000 ng/ml to 100 pg/ml. PCR efficiencies and relative expression levels of GADD153 and BiP as a function of eEF1*α*1 expression were calculated as previously described (34).

### Western Blot Analysis—

LS8 cells were plated in 10-cm dishes and treated with 2 mm NaF or 0.5 *μ*g/ml tunicamycin (Sigma) for 24 h. Nuclear proteins were extracted by resuspending washed cells in harvest buffer (10 mm HEPES, pH 7.9, 50 mm NaCl, 0.5 m sucrose, 0.1 mm EDTA, 0.5% Triton X-100, 1 mm dithiothreitol, 10 mm tetrasodium pyrophosphate, 100 mm NaF, 17.5 mm *β*-glycerophosphate, 1 mm phenylmethylsulfonyl fluoride, 4 *μ*g/ml aprotinin, and 2 *μ*g/ml pepstatin A). After centrifugation, the precipitate was resuspended in buffer (10 mm HEPES, pH 7.9, 500 mm NaCl, 0.1 mm EDTA, 0.1 mm EGTA, 0.1% Nonidet P-40, 1 mm dithiothreitol, 1 mm phenylmethylsulfonyl fluoride, 4 *μ*g/ml aprotinin, and 2 *μ*g/ml pepstatin A). Insoluble debris was removed by centrifugation, and the protein concentration of nuclear extracts was determined by the BCA protein assay kit (Pierce). Thirty *μ*g of protein was run on 12.5% SDS-PAGE gels and transferred to nitrocellulose membranes (Bio-Rad). Antibodies (Santa Cruz Biotechnologies) were specific for GADD153 (sc-7351, 1:2000) or Xbp-1 (M-186, 1:3000). After incubation with appropriate primary and horseradish peroxidase-conjugated secondary antibodies (anti-mouse IgG, 1:5000; anti-rabbit IgG, 1:5000, Cell Signaling Technology), specific protein bands were detected and analyzed by enhanced chemiluminescence substrate detection (ECL Western blotting analysis system, Amersham Biosciences).

### Immunocytochemistry—

LS8 cells were plated in a four-well chamber slide (BD Biosciences). Cells were treated with or without 2 mm NaF for 24 h and fixed with 4% paraformaldehyde in phosphate-buffered saline. Primary antibody was either mouse monoclonal anti-GADD153 (sc-7351; Santa Cruz) or antisera specific for Ire1.^2^ The Vector M.O.M. immunodetection kit (Vector) was used to detect GADD153, and the VectaStain Elite ABC kit (Vector) was used to detect Ire1. The staining procedure was performed according to the manufacturer’s instructions.

### Inhibition of GADD153 with Short Interfering RNA (siRNA)—

siRNA was used to down-regulate GADD153 expression (Qiagen). The siRNA sequence was AACAGAGGTCACACGCACATC. Briefly, LS8 cells were plated in 10-cm dishes (12 ml of medium without antibiotics) and transiently transfected with 0.4 *μ*m siRNA in 3 ml of Opti-MEM with 30 *μ*l of Lipofectamine 2000 (Invitrogen). For 96-well plates LS8 cells were plated at 100 *μ*l/well and transiently transfected with 0.4 *μ*m siRNA in 100 *μ*l of Opti-MEM with 0.25 *μ*l of Lipofectamine 2000. After incubation for 24 h, cells were treated with or without 5 mm NaF for 24 h. Nuclear proteins were extracted as described above and used for Western blot or DNA fragmentation analysis.

### Overexpression of GADD153—

The mouse GADD153 cDNA was present or not (control) in the pcDNA3 vector (Invitrogen). The plasmids were transfected using Lipofectamine 2000 (Invitrogen). GADD153 protein expression was detected by Western blot analysis. Five *μ*g of protein from whole cells were subjected to SDS-PAGE. In brief, cells were plated in 6-well plates and transiently transfected with 0.75 or 1.5 *μ*g of DNA in 0.5 ml of Opti-MEM with 5 *μ*l of Lipofectamine 2000. After 24 h cells were collected and lysed (50 mm Tris-HCl, pH 8.0, 150 mm NaCl, 1% Nonidet P-40, 0.5% sodium deoxycholate, 0.1% SDS, 50 mm NaF, 1 mm EDTA, 0.1% protease inhibitor mixture, 0.5 mm phenylmethylsulfonyl fluoride). Insoluble debris was removed by centrifugation, and the protein concentration was determined by the BCA protein assay kit (Pierce). DNA fragmentation analysis was performed as described above.

### In Vivo TUNEL Assay and Immunohistochemistry—

All animals used in this work were housed in AAALAC-approved facilities, and all operations were performed in accord with protocols approved by the Institutional Animal Care and Use Committees at the Forsyth Institute. Six-week-old male C57BL/6J mice were purchased from Charles River Laboratories. Fluoride at a concentration of 0, 75, or 150 ppm as NaF was delivered *ad libitum* in the drinking water for 3–4 weeks. Fluoride concentration analyses of chow were performed in duplicate by a modification (36) of the hexamethyldisiloxane (Sigma) microdiffusion method, and serum fluoride levels were performed by the method of Vogel *et al*. (37). Incisors were formalin-fixed, paraffin-embedded, and sectioned. For the TUNEL assay, the In Situ Cell Death Detection kit (Roche Applied Science) was used according to the manufacturer’s instructions. The sections were incubated with anti-fluorescein antibody conjugated with horseradish peroxidase. For immunohistochemistry, sections were incubated in blocking agent (goat serum) for 20 min, in active Ire1*α*-specific antisera (1:100) overnight, in peroxidase-conjugated antibody (Vectastain Elite Reagent), and in Sigma Fast 3,3′-diaminobenzidine substrate. Sections were examined by light microscopy for the presence of fragmented DNA and for the presence of active Ire1*α*.

## RESULTS

### NaF Inhibits LS8 Cell Growth—

**Fig. 1.**
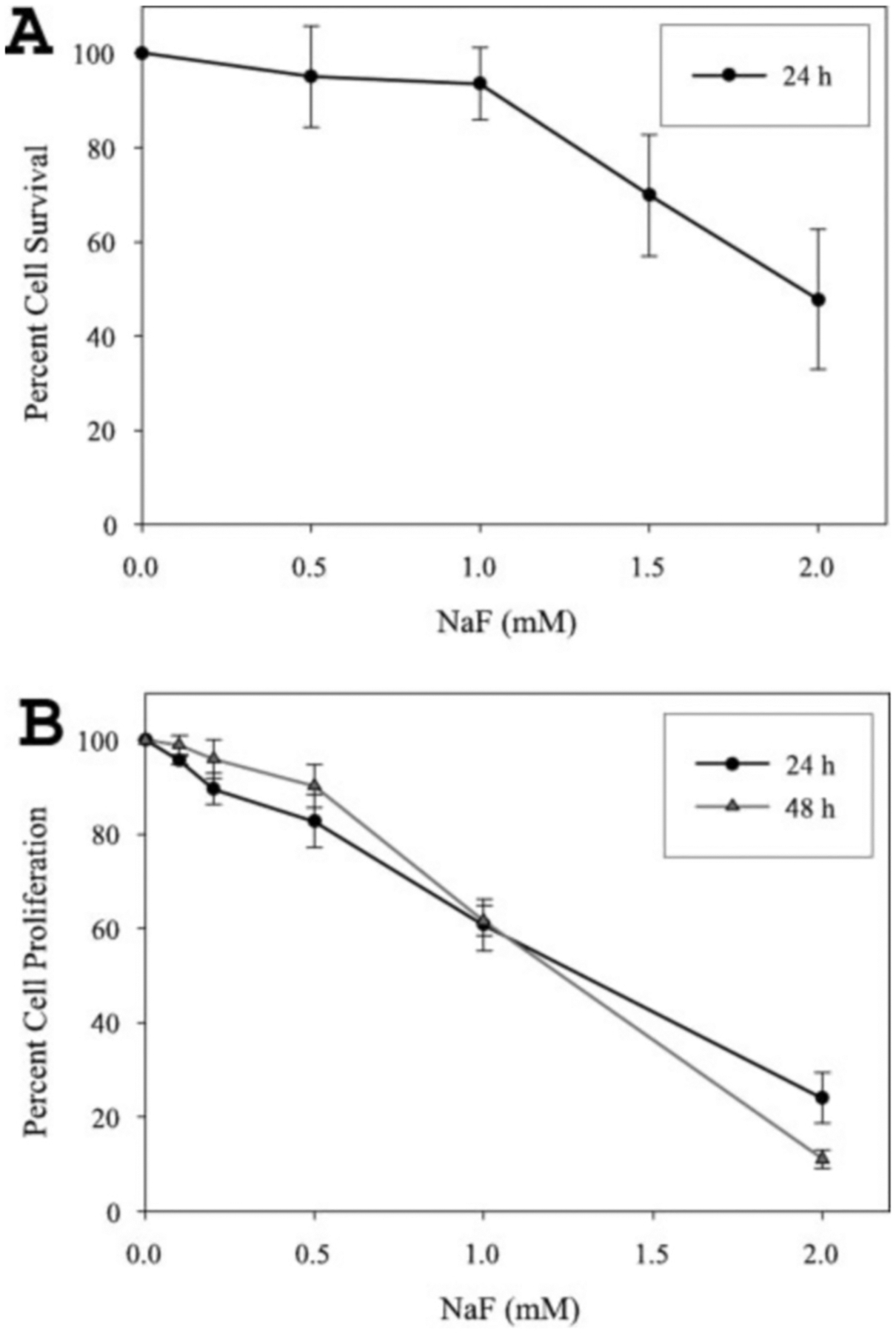
Toxic and antiproliferative effects of NaF treatment on LS8 cells. *A*, limiting dilutions of LS8 cells were seeded into culture flasks, allowed to adhere for 18 h, and treated for 24 h with the indicated concentrations of NaF. After 8–9 days, the resulting colonies were stained and counted, and percent cell survival was calculated (number treated/untreated colonies) × 100. Colonies from three flasks were counted for each experimental treatment group, and three separate experiments were performed. *Error bars* represent the S.D. *B*, LS8 cells were seeded into 96-well plates and treated for either 24 or 48 h with the indicated concentrations of NaF. Reduction of MTT to an insoluble formazan dye by mitochondrial enzymes was quantified for each well by *A*_550_ measurements, and the results were used to calculate percent cell proliferation (treated *A*_550_**/**untreated *A*_550_ × 100). Six wells were assayed for each experimental treatment, and three separate experiments were performed. *Error bars* represent the S.D.

To establish the concentration of NaF necessary for toxicity of the ameloblast-derived LS8 cell line, we performed cell survival assays. LS8 cells were seeded into 25-cm^2^ flasks (1000 cells/flask) for 18 h before 24 h of treatment with NaF at concentrations of 0, 0.5, 1.0, 1.5, and 2.0 mm. After 8–9 days, the resulting colonies were stained with methylene blue and counted. Experiments were performed in triplicate, and colony counts from the treatment groups were compared with the untreated controls (Fig. 1A). Although the trend started at the lowest dose assayed (0.5 mm), significant levels of cell death were observed at the 1.5 (30%) and 2.0 mm (52%) NaF concentrations (Fig. 1A).

Next we quantified LS8 cell proliferation by use of the tetrazolium salt MTT. MTT is reduced to an insoluble formazan dye by mitochondrial enzymes associated with metabolic activity, and the amount of dye formed correlates positively to the number of proliferating cells present. LS8 cells were treated with several concentrations of sodium fluoride of between 0 and 2 mm for either 24 or 48 h followed by assessment of cell proliferation by the MTT assay. Treatment with 0.1 mm NaF (1.9 ppm fluoride) reduced LS8 cell proliferation by about 4%, and a significant reduction in cell proliferation (~10%) was observed after exposure to 0.2 mm NaF (3.8 ppm fluoride) for 24 h (Fig. 1B). After 48 h of fluoride treatment, the cells appeared to have time to recover from the low dose exposure (0.1, 0.2, and 0.5 mm) and proliferate at a slightly higher rate than the 24-h treatment groups. However, at the highest dose (2.0 mm), significantly less proliferation was apparent for the 48-h treatment compared with the 24-h treatment (Fig. 1B).

### NaF Induces Caspase-mediated DNA Fragmentation in LS8 Cells—

**Fig. 2.**
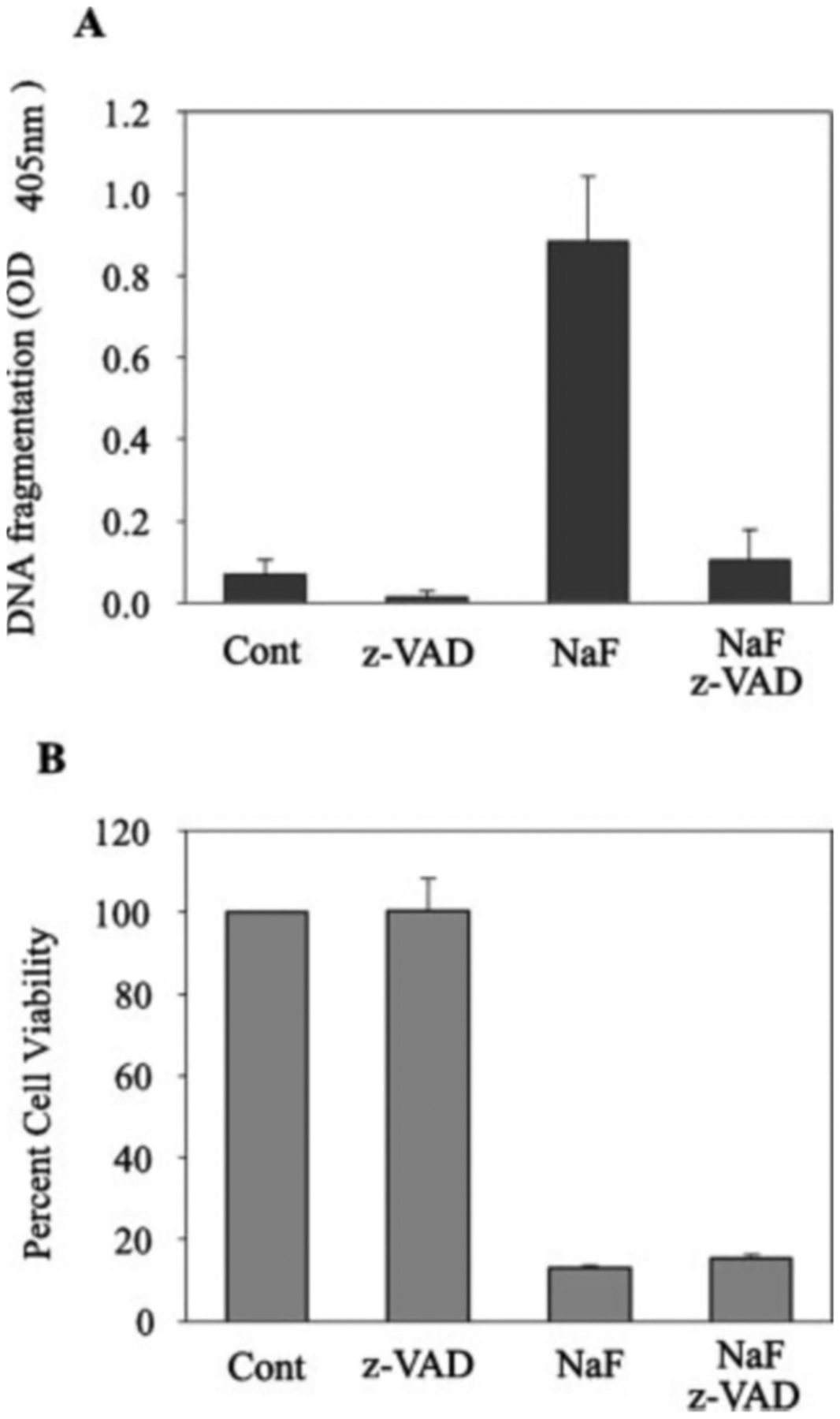
Role of caspases in NaF induced LS8 cell death. Cells were pretreated or not with 50 *μ*m z-VAD for 1 h followed by 48 h of treatment with 2 mm NaF. *A*, adherent cells were assayed for DNA fragmentation by an ELISA-based TUNEL assay that quantifies DNA strand breaks by measuring bound peroxidase activity (*A*_405_). Two wells were assayed for each experimental treatment, and three separate experiments were performed. *Error bars* represent the S.D. Note that treatment of LS8 cells with NaF generated significant levels of DNA fragmentation that were almost completely eliminated by pretreatment with z-VAD. *Cont*, control. *B*, trypan blue dye exclusion was performed to obtain numbers of live cells after NaF treatment. Percent viability was calculated by counting live cells (number treated/number untreated × 100). Two wells of a 24-well plate were analyzed for each experimental treatment, and 3 separate experiments were performed. *Error bars* represent the S.D. Note that z-VAD did not significantly protect LS8 cells from the toxic effects of 2 mm, 48 h NaF treatment.

DNA fragmentation was quantified by use of an ELISA-based TUNEL assay where adherent cells were assayed for DNA strand breaks. After 48 h of treatment with 2.0 mm NaF, a significant quantity of LS8 DNA strand breaks were observed (Fig. 2A). The addition of the general caspase inhibitor z-VAD eliminated the NaF-induced DNA strand breaks, demonstrating that caspases mediated the DNA fragmentation response. To determine whether z-VAD treatment protected the cells from NaF-induced cell death, we performed trypan blue dye exclusion assays in duplicate for each treatment in three different successive experiments (Fig. 2B). The results demonstrated that z-VAD did not significantly protect LS8 cells from the toxic effects of NaF, indicating that caspases are involved but are not essential for NaF-induced cell death.

### NaF Induces ER Stress—

**Fig. 3.**
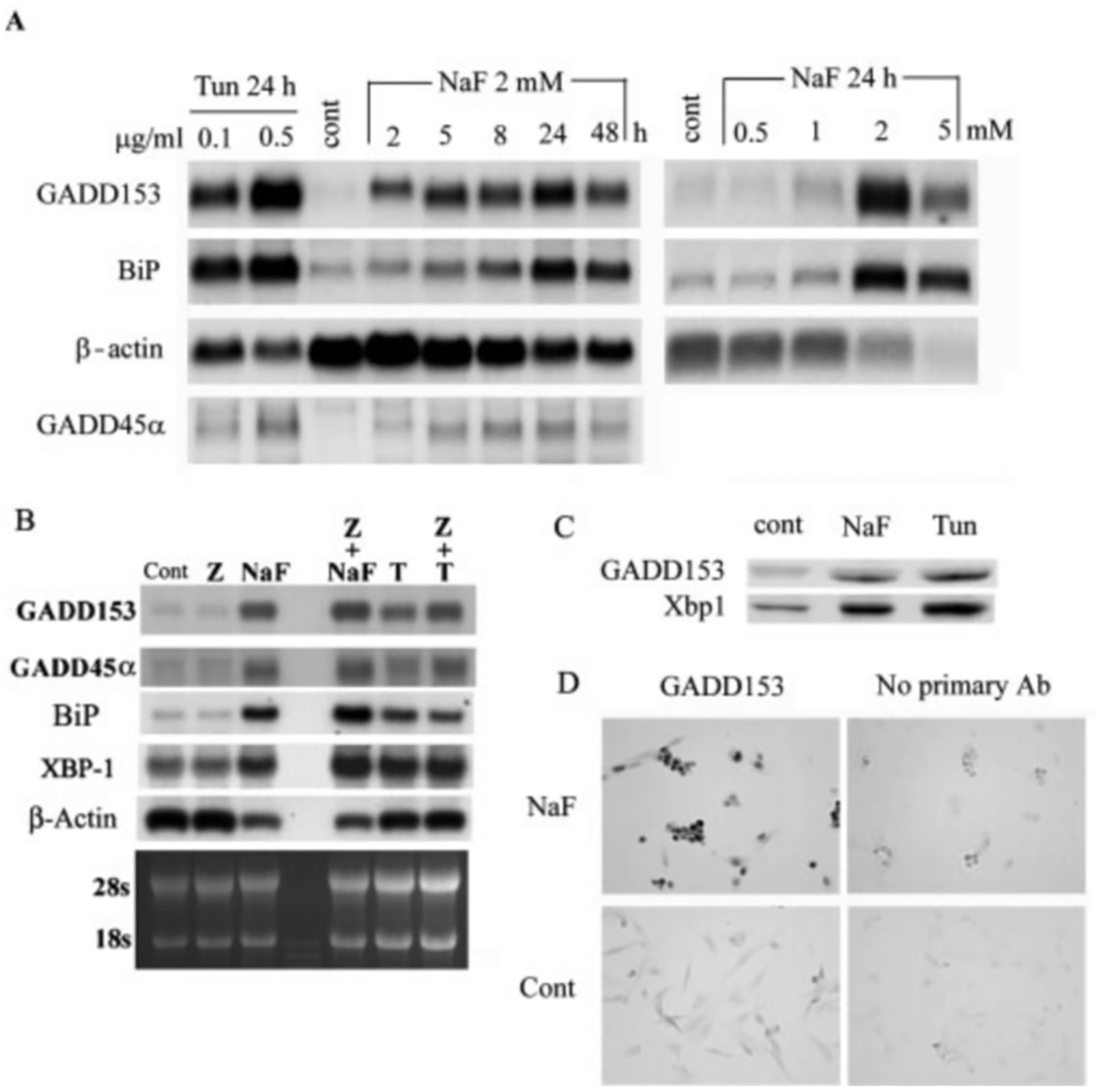
Treatment of LS8 cells with NaF induces the ER stress response. *A*, representative Northern blots with 10 *μ*g total RNA/lane. Treatment times and doses are indicated *above* each *lane*, and the identity of the mRNA assayed is listed *beside* each *blot*. The same blot was stripped and reprobed for each mRNA assayed. Treatment of LS8 cells with tunicamycin (*Tun*) served as the ER stress response positive control (*cont*). With the exception of the *β*-actin control, the stress response genes assayed displayed increased expression in a time- and dose-dependent manner after NaF treatment. *B,* Northern blot analysis of LS8 gene expression as a function of pretreatment with z-VAD (*Z*). The same filter was stripped and reprobed for each mRNA assayed. Tunicamycin (*T*) served as the positive control. Note that the absence of DNA fragmentation did not affect NaF-mediated gene transcription. *C*, Western blot of protein (30 *μ*g) isolated from LS8 cell nuclei after 24 h of treatment with NaF (2 mm) or tunicamycin (0.5 *μ*g/ml). Both GADD153 and the active form of Xbp1 were induced by each treatment. *D*, immunohistochemical staining of LS8 cell nuclei with a monoclonal antibody specific for GADD153. Cells were treated or not with 2 mm NaF for 24 h as indicated. *Panels* to the *left* were stained with both primary and secondary antibodies (*Ab*). Panels to the right were stained with secondary antisera only. Immunolabeling detected strong expression of GADD153 in the NaF-treated cells.

Because LS8 cells are more sensitive to the antiproliferative rather than the toxic effects of NaF, we asked if NaF induced a cell stress response. GADD genes are induced by growth arrest and DNA damage, so we assessed the expression levels of two GADD genes before and after NaF treatment. Northern blot analysis demonstrated that both GADD153 and GADD45*α* mRNAs were induced in LS8 cells after NaF exposure. The GADD153 induction was the strongest and increased in a time- and dose-dependent manner that peaked (40–60-fold induction) after 24 h of 2 mm NaF exposure (Fig. 3A). Because GADD153 expression may or may not stem from the ER stress response pathway (26, 27), we asked if the ER stress response gene BiP was also induced by exposure to NaF. BiP is an ER resident molecular chaperone that is thought to prevent protein aggregation while maintaining a protein-folding-competent state (38). BiP expression was increased in a time- and dose-dependent manner (Fig. 3A) and also peaked at 24 h of 2 mm NaF treatment (20-fold induction).

To determine whether the NaF-elicited response in LS8 cells was similar to that elicited by a verified ER stress inducer, we examined mRNA levels of GADD153, GADD45*α*, and BiP after treatment with tunicamycin. Tunicamycin induces ER stress by inhibiting *N*-linked protein glycosylation and has been demonstrated to induce GADD153 and BiP expression in other cell lines (39). Treatment of LS8 cells with 0.1 or 0.5 *μ*g/ml tunicamycin for 24 h significantly induced GADD153 expression by 50- or 100-fold and induced BiP expression by ~17- or 27-fold, respectively (Fig. 3A). GADD45*α* was also induced, but similar to the NaF treatments, the overall level of the mRNA present was substantially less than that of GADD153 or BiP.

Because GADD genes are induced by growth arrest and DNA damage, we asked if caspase-mediated DNA fragmentation was a necessary component of the ER stress response. LS8 cells were pretreated or not with 50 *μ*m z-VAD followed by treatment for 48 with either 2 mm NaF or 0.1 *μ*g/ml tunicamycin. The induction of GADD153, GADD45*α*, BiP, and XBP-1 message was not altered by prior treatment with z-VAD (Fig. 3B), indicating that the gene products function upstream of the DNA damage caused by caspase activation.

Next we asked if the increased NaF-induced mRNA levels translated into increased levels of GADD153 protein and the active form of XBP-1. ER stress can initiate splicing of XBP1 mRNA so that the translation reading frame becomes shifted to encode a potent leucine-zipper-containing transcription factor. This transcription factor can bind to the ERSE to activate various ER stress response genes (21). Therefore, we performed Western blots with antisera specific for GADD153 and Xbp1 to determine whether NaF treatment induced their expression. NaF treatment (2 mm, 24 h) increased the protein levels of GADD153 and of the active form of Xbp1 (Fig. 3C). Immunocytochemical analysis of LS8 cells confirmed the NaF-mediated increase in GADD153 expression (Fig. 3D). Strong staining was observed in the NaF-treated cells, but staining remained low or undetectable in untreated cells and in cells treated with NaF and the secondary antibody only. These data demonstrate that NaF treatment induces the ER stress response pathway in LS8 cells.

### NaF Induces ER stress in First Passage Enamel Organ (EO) cells—

To confirm the results demonstrated in LS8 cells for non-transformed cells extracted from the EO, unerupted third molars were removed from pig mandibles and dissociated into cell suspensions (33). Suspensions were then passed through a 40-*μ*m filter to collect single cells. The cells were allowed to grow and were treated (experimental) or not (control) with 2 mm NaF for 48 h. EO cells were collected, and total RNA was extracted for quantitative real-time PCR analysis. Before the analysis several housekeeping and internal control genes were tested for their ability to maintain stable expression during NaF treatment. Pig eEF1*α*1 was the most stably expressed and served as the internal control mRNA for the quantification of GADD153 and BiP expression. Both the GADD153 and BiP mRNAs were induced substantially by exposure to NaF (Table I). Thus, in addition to the LS8 enamel organ-derived cell line, ER stress was also demonstrated in first passage EO cells that were exposed to NaF.

### NaF Activates the Expression of Intracellular Carbonic Anhydrase—

**Fig. 4.**
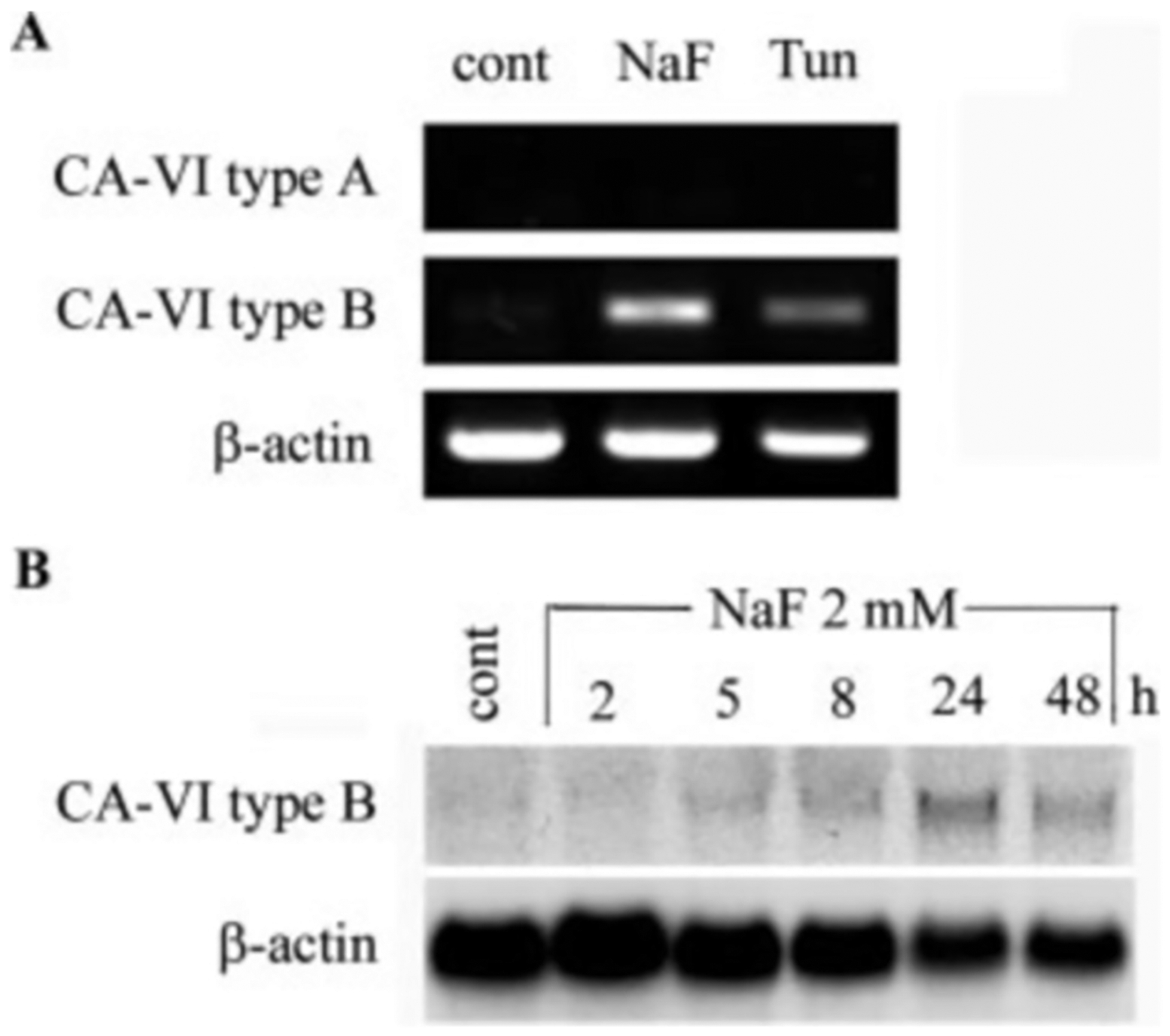
Treatment of LS8 cells with NaF activates the expression of CA-VI type B. *A*, reverse transcription-PCR analysis for identification of CA-VI mRNA splice variant. Primers specific for type A (encodes a signal sequence) or type B (encodes no signal sequence) demonstrated the activation of only the type B variant after treatment with NaF (2 mm, 24 h) or tunicamycin (*Tun*, 0.1 *μ*g/ml, 24 h). *cont*, control. *B*, Northern blot (10 *μ*g of total RNA/lane) time-course analysis of CA-VI type B expression. Expression peaked after 24 h of 2 mm NaF exposure.

VI (CA-VI)-GADD153 was previously demonstrated to induce CA-VI expression (40). This CA-VI was demonstrated as a novel intracellular form (type B) rather than the standard secreted CA-VI form (type A) (41). Because natural ameloblast cells of the enamel organ are exposed to large amounts of acid as the hydroxyapatite that enamel is composed of grows, we asked if CA-VI was also induced in LS8 cells by NaF treatment. To determine whether type A or type B CA-VI expression was induced, we performed diagnostic reverse transcription-PCR analysis. Each primer set was specific for either the type A or type B form. The results demonstrated that both NaF (2 mm, 24 h) and tunicamycin (0.1 *μ*g/ml, 24 h) activated the expression of CA-VI type B (Fig. 4A). No expression of the type A form was evident in LS8 cells regardless of the presence or absence of the ER stress inducers (Fig. 4A). The NaF-induced CA-VI PCR band was purified and sequenced to confirm its identity. Northern blot time-course analysis demonstrated that CA-VI expression was activated to peak levels after 2 mm NaF treatment for 24 h (Fig. 4B).

### Attenuation of GADD153 Expression Increases DNA Fragmentation in LS8 Cells, Whereas Transient Overexpression Has Little Effect—

**Fig. 5.**
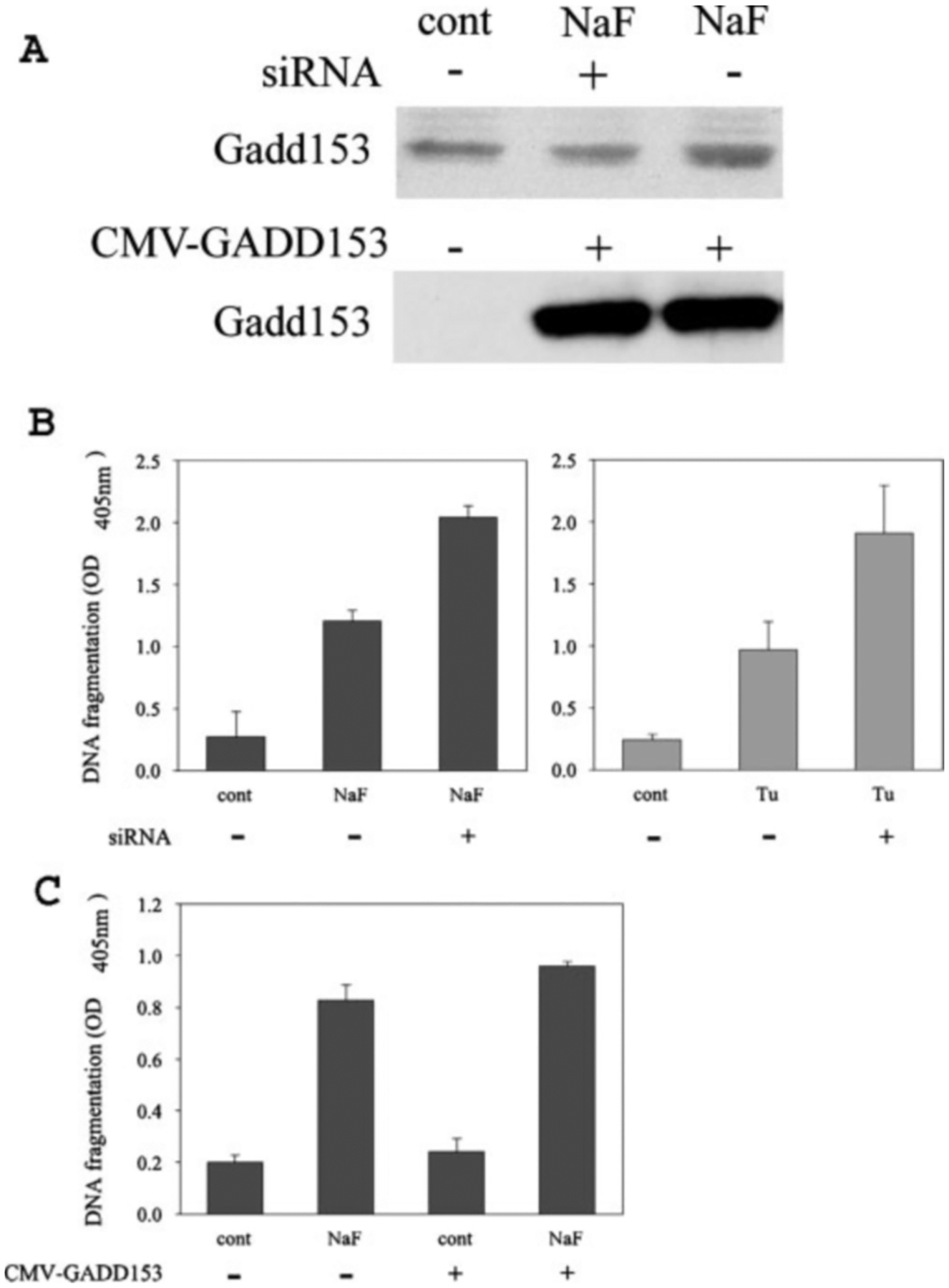
Attenuation of GADD153 expression increases NaF-induced DNA fragmentation in LS8 cells, whereas transient overexpression has little effect. *A*, representative Western blots of GADD153 expression. *Top*, pretreatment with GADD153 siRNA (*NaF*, +) reduced the NaF-induced GADD153 up-regulation (*NaF*, −) to levels approximately equivalent to control cultures pretreated with nonspecific siRNA-only (*cont*, −). Each lane contains 30 *μ*g nuclear protein (*top*). Transient transfection of either 0.75 *μ*g (+, *middle lane*) or 1.5 *μ*g (+, *right lane*) CMV-GADD153 vector dramatically increased GADD153 expression over that of cells treated with empty CMV vector alone (−, *left lane*). Each lane contains 5 *μ*g of whole cell protein (*bottom*). *B*, ELISA-based TUNEL assays for quantification of DNA strand breaks. NaF-induced DNA fragmentation of cells pretreated with GADD153 siRNA (*NaF*, +) demonstrated significantly more strand breaks (*left panel*) than those treated with nonspecific siRNA and NaF (*NaF*, −) or with siRNA alone (*cont*, −). Results similar to the NaF treatments were obtained with 0.1 *μ*g/ml tunicamycin (*Tu*) treatment for 24 h (*right panel*). *C*, ELISA-based TUNEL assay for quantification of DNA strand breaks. Cells were transfected with either empty CMV vector (−) or CMV-GADD153 vector (+) and were untreated (*cont*) or were treated with NaF (*NaF*). Regardless of NaF treatment, GADD153 overexpression had little to no effect on DNA fragmentation. All NaF treatments were 5 mm for 24 h, and for all TUNEL assays, two wells of a 96-well plate were analyzed for each experimental treatment, and three separate experiments were performed. *Error bars* represent the S.D.

siRNA was designed and used to reduce the levels of LS8 GADD153 mRNA. Western blot analysis confirmed that GADD153 siRNA treatment reduced the amount of GADD153 protein expressed by NaF-treated LS8 cells (Fig. 5A). The cells were then assessed for levels of DNA fragmentation. Cells treated with nonspecific control siRNA and NaF or tunicamycin exhibited significant amounts of DNA fragmentation. However, when cells were treated with GADD153 siRNA, the amount of DNA fragmentation observed after NaF or tunicamycin treatment was nearly doubled, suggesting that GADD153 plays a role in preventing DNA fragmentation (Fig. 5B). To further explore this possibility, we transiently overexpressed GADD153 in LS8 cells by transfecting them with a CMV-driven expression plasmid (CMV-GADD153). Western blot analysis confirmed that a dramatic increase of GADD153 protein level occurred as a result of the transfection (Fig. 5A). NaF-induced (5 mm, 24 h) DNA fragmentation was assessed after transfection with empty expression vector (CMV), CMV transfection followed by NaF treatment, CMV-GADD153 transfection, and CMV-GADD153 transfection followed by NaF treatment (Fig. 5C). Interestingly, GADD153 overexpression did not significantly alter LS8 DNA fragmentation in the presence or absence of NaF.

### Caspase-3 Is Responsible for Increased DNA Fragmentation In GADD153^−/−^ Cells—

**Fig. 6.**
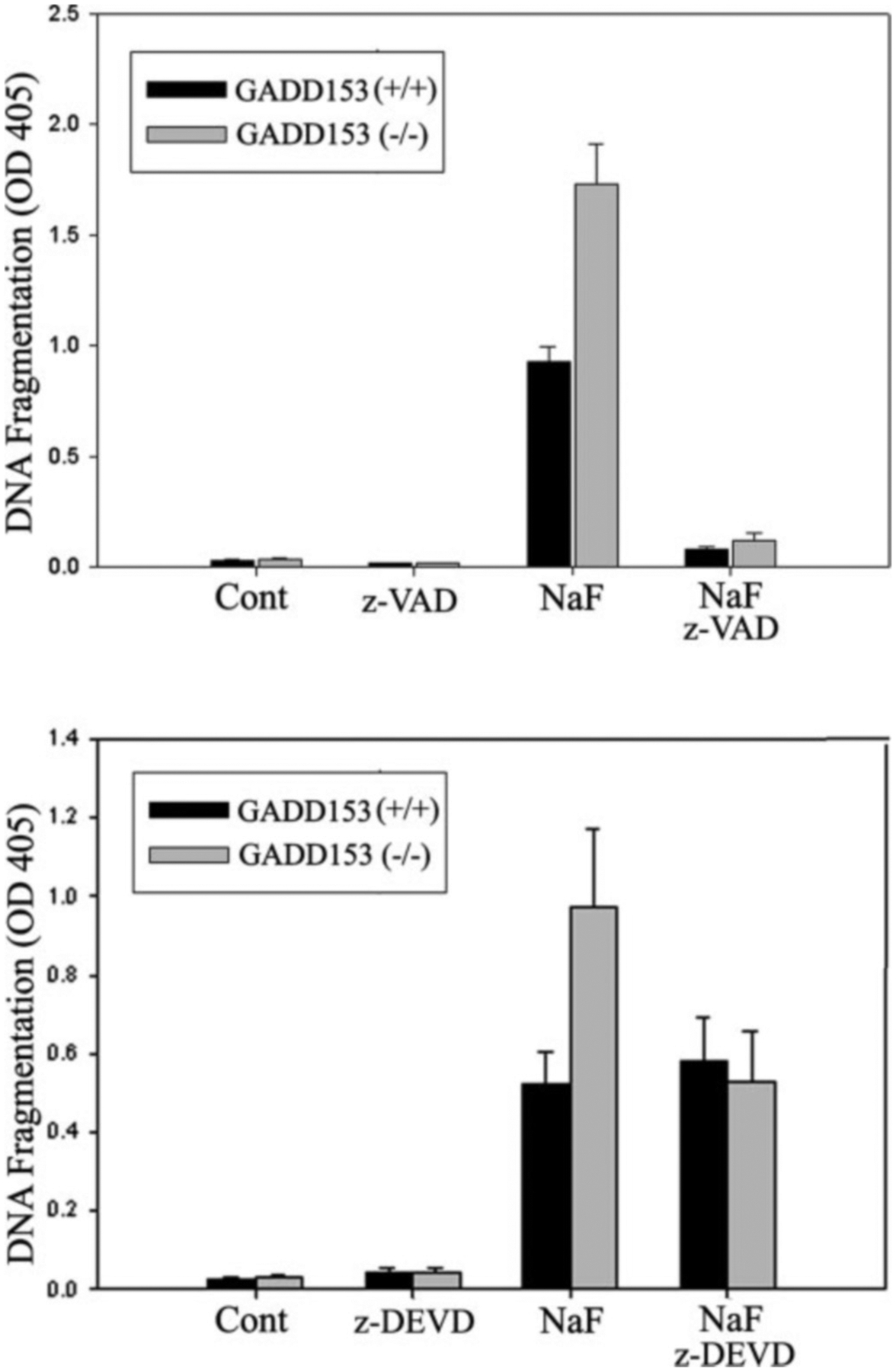
Caspase-3 activity is responsible for increased levels of NaF-mediated DNA fragmentation in GADD153^−/−^ cells *versus* controls. Mouse embryonic fibroblasts with (GADD153^−/−^) or without (GADD153^+/+^) homozygous GADD153 gene deletion were pretreated or not with either 50 *μ*m z-VAD (*top panel*) or 2 *μ*m z-DEVD (*bottom panel*) for 1 h followed by 24 h of treatment with 5 mm NaF. Adherent cells were assayed for DNA fragmentation by the ELISA-based TUNEL assay. Three wells were assayed for each experimental treatment, and three separate experiments were performed. *Error bars* represent the S.E. of the mean. Note that significantly more NaF-induced strand breaks occurred in the GADD153^−/−^ cells than in the control cells and that inhibition of caspase-3 with z-DEVD eliminated this difference. *Cont*, control.

To confirm and further explore the role of GADD153 in caspase-mediated DNA fragmentation, we assessed fragmentation in SV40 T-antigen-transformed mouse embryonic fibroblasts (MEF). The MEF cells were either GADD153^+/+^ or had homozygous deletion of the GADD153^−/−^ gene. As demonstrated for LS8 cells, treatment of MEF cells with NaF did result in significantly increased levels of caspase-mediated DNA fragmentation that were virtually eliminated by treatment with the pan-caspase inhibitor z-VAD. The adherent GADD153^−/−^ cells had nearly twice as much fragmentation as did the adherent GADD153^+/+^ cells (Fig. 6, *top panel*). Because NaF was previously demonstrated to activate caspase-3 (42), we assessed the role of caspase-3-mediated DNA fragmentation in MEF cells by use of the caspase-3 inhibitor z-DEVD. Pretreatment of MEF cells with 2 *μ*m z-DEVD significantly reduced GADD153^−/−^ DNA fragmentation to levels similar to those of the GADD153^+/+^ cells. However, z-DEVD treatment did not significantly alter the fragmentation levels observed in the GADD153^+/+^ cells. These results suggest that in adherent cells GADD153 may lead to the inhibition of caspase-3-mediated DNA fragmentation.

### Characterization of Mouse Incisor Enamel Organ after Exposure to Fluoridated Drinking Water—

Mice were exposed to 75 or 150 ppm fluoride as NaF in the drinking water *ad libitum* to determine whether active Ire1 was induced and if their enamel organs were adversely affected. Mouse incisors continuously erupt so the incisor enamel organs are present throughout adulthood. After 3–4 weeks of NaF treatment mice were bled and sacrificed, and their white fluorotic incisors were immediately dissected and demineralized for sectioning. Fluoride concentrations in mouse chow averaged 20 *μ*g/g (20.4 ppm), and mouse serum fluoride concentrations averaged: 0 ppm fluoride, 0.18 *μ*g/ml; 75 ppm fluoride, 0.29 *μ*g/ml; 150 ppm fluoride, 0.37 *μ*g/ml (Table II).

**Fig. 7.**
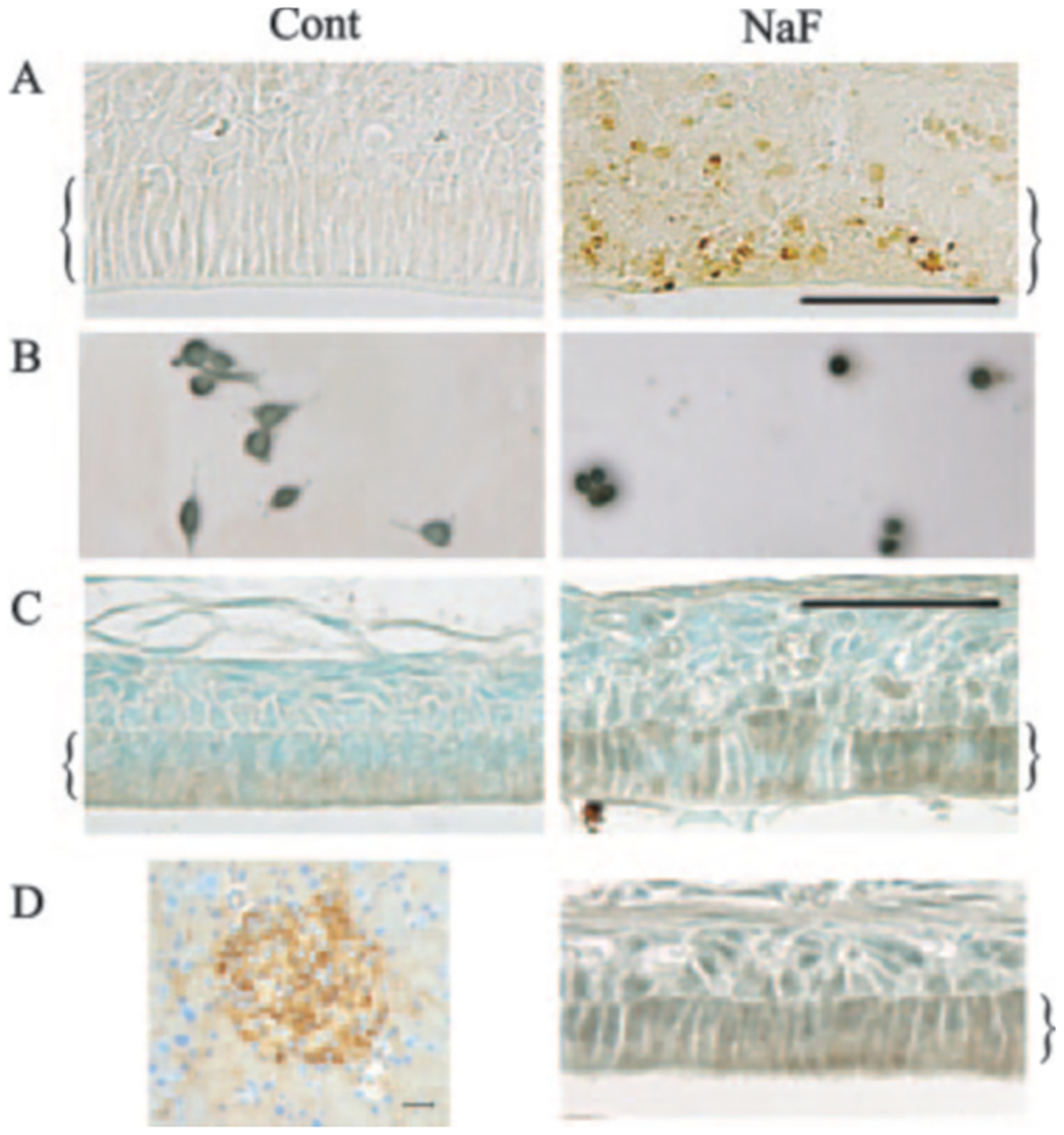
High dose fluoride in drinking water causes increased enamel organ apoptosis and apparent induction of Ire1. *A*, *in situ* TUNEL assay performed after 3–4 weeks of exposure to 150 ppm fluoride delivered *ad libitum* in drinking water. Untreated control secretory stage enamel organ with healthy columnar-shaped ameloblasts (*left panel*, *bracket*). Fluoride-treated secretory stage enamel organ (*right panel*). Note that the fluoride-treated enamel organ morphology was disrupted since no healthy ameloblasts can be observed. Also, several apoptotic cells were observed where the ameloblasts are normally located (*right panel*, *bracket*). *Cont*, control. *B,* LS8 cells were treated (*right panel*) or not (*left panel*) with 2 mm NaF for 24 h and processed and stained by immunohistochemical methods for the presence of active Ire 1. Note that staining was more intense in the rounded NaF treated cells when compared with the oval untreated cells. *C*, immunohistochemical staining for active Ire1 after 3–4 weeks of exposure to 75 ppm fluoride delivered *ad libitum* in drinking water. Shown is untreated maturation stage ameloblasts demonstrating low level staining for active Ire1 (*left panel*, *bracket*). Fluoride treated ameloblasts demonstrating increased staining for Ire1 (*right panel*, *bracket*). Note that some ameloblasts stained more strongly than others (*bars*, 50 *μ*m). *D*, immunohistochemical staining of pancreatic islet cells with the same antisera specific for active Ire1 (*left panel*). Another mouse incisor section demonstrating active Ire1 staining after 3–4 weeks of exposure to 75 ppm fluoride delivered *ad libitum* in drinking water is shown.

For the 150-ppm-treated mice, sections were assessed by TUNEL assay for the presence of nuclei containing fragmented DNA. The enamel organs from these mice had lost their distinctive morphology. In contrast to controls, no columnar ameloblasts cells were present in the NaF-treated enamel organs (Fig. 7A). TUNEL staining revealed that the NaF-treated mice contained more positively stained nuclei than controls, especially in areas where ameloblasts were normally observed. We next asked if NaF treatment induced the dimerized active form of Ire1. LS8 cells were assessed for active Ire1 by immunocytochemical means. Cells treated with 2 mm NaF for 24 h did display stronger staining when compared with the untreated control cells (Fig. 7B), indicating that Ire1 had become activated by the NaF exposure. To determine whether Ire1 was active *in vivo* in normal mouse ameloblasts or in ameloblasts where mice were exposed to 75 ppm fluoride in drinking water, Ire1 immunostaining was also performed. Both the controls and fluoride-treated mice had ameloblasts that stained positively for Ire1, and it appeared that the staining was more intense for the fluoride-exposed mice (Fig. 7, *C* and *D*, *right panel*). Pancreatic *β*-cells secrete large quantities of insulin and have a naturally induced ER stress response to accommodate the protein load.^2^ Therefore, as a positive control we tested the antisera specific for activated IRE1 on *β*-cells present within the mouse pancreas. As expected, strong staining was observed in the *β*-cells but not in the surrounding tissue (Fig. 7D, *left panel*). These results suggest that normal mouse ameloblasts may undergo an ER stress response that is enhanced by exposure to high dose fluoride.

## DISCUSSION

Fluoride-induced ER stress was demonstrated after NaF treatment of both the ameloblast-like LS8 cell line and first passage porcine EO cells. For LS8 cells, fluoride exposure significantly reduced cell growth, decreased cell survival, induced caspase-mediated DNA fragmentation, and induced the expression of GADD153, GADD45*α*, BiP, the active form of Xbp-1, and the non-secreted form of CA-VI. Although NaF induction of the UPR has not been demonstrated previously, prior published results are consistent with this result. For example, ER stress may cause an overall reduction in protein synthesis so that the cell can cope with the existing unfolded or misfolded proteins (43). As early as the mid-1960s, NaF exposure was demonstrated to inhibit protein synthesis in several different cell and tissue types (44). Specifically, treatment of rats with 75 or 100 ppm fluoride in drinking water caused a reduction in enamel protein synthesis and a reduction of enamel protein removal from the matrix (45). Another report demonstrated that after NaF exposure, rat incisor ameloblasts had a disturbance of vesicular transport between the ER and Golgi apparatus (46). Also, the apical ends of ameloblasts cycle between a ruffle-ended and smooth-ended morphology during the maturation stage of enamel development, and 100 ppm NaF in rat drinking water will delay this cycle by as much as 30% (47). These previous results are consistent with fluoride-induced ER stress with subsequent induction of the UPR.

Prior studies have demonstrated that NaF may induce apoptosis in cultured cells (4, 42, 48–52). Our results demonstrating that NaF treatment induces apoptosis are in agreement with these previous studies. To determine whether NaF-induced DNA strand breaks in LS8 cells, we used a sensitive ELISA assay that detects cleaved nucleosomal DNA. Only adherent cells were assayed for each treatment group in order to avoid assaying nonspecific necrotic DNA fragmentation. Effector caspases activate the DNA fragmentation factor, which cleaves DNA between nucleosomes. However, DNA fragmentation proved dispensable for LS8 cell death because, although z-VAD treatment prevented fragmentation, it did not protect the LS8 cells from NaF-induced toxicity (Fig. 2). Previously it was demonstrated that deletion of DNA fragmentation factor from the mouse genome dramatically reduces apoptotic DNA fragmentation but does not alter developmentally regulated apoptotic events. These mice breed and develop normally (53). Although it has been suggested that DNA fragmentation serves as a means to eliminate inserted viral DNA (54), it was dispensable for normal developmentally programmed cell death just as it was dispensable for NaF-induced LS8 cell death.

Previously, GADD153 was demonstrated to induce the expression of a non-secreted form of carbonic anhydrase VI (CA-VI type B) (41). Carbonic anhydrase catalyzes the reversible hydration of CO_2_ by combining CO_2_ and H_2_O to form bicarbonate ions (${\text{HCO}}_{3}^{-}$) and hydrogen (H^+^) ions (55). This is a potentially important reaction for ameloblasts, because during the maturation stage of development apatite crystals in enamel grow at their most rapid rate (56). This results in the creation of excessive quantities of H^+^ ions ($10\;{\text{Ca}}^{2+}+6\;{\text{HPO}}_{4}^{2-}+2\;{\text{H}}_{2}\text{O}\to {\text{Ca}}_{10}{\left({\text{PO}}_{4}\right)}_{6}{\left(\text{OH}\right)}_{2}+8\;{\text{H}}^{+}$), which overwhelm the local buffering capacity of the tissue fluids that percolate through the developing enamel (57). To compensate for this, it has been proposed that ameloblasts, which form a relatively tight permeability barrier at the enamel surface, excrete large amounts of ${\text{HCO}}_{3}^{-}$ ions into the enamel by way of a band 3-type anionic exchanger (57, 58). Thus, mechanisms are in place that depend on cytoplasmic carbonic anhydrase activity to keep pH of the developing enamel layer from becoming too acidic. The expression of the non-secreted form of CA-VI was induced to its highest level in LS8 cells 24 h after NaF treatment (Fig. 4). Whether or not the non-secreted form of CA-VI plays a role in enamel formation remains to be explored. However, maintenance of pH does appear to be a priority during ER stress when, in general, protein synthesis is otherwise reduced.

The results demonstrating that NaF and tunicamycin-induced DNA fragmentation increased upon inhibition of GADD153 levels (Fig. 5) was unexpected. We further characterized these results by assessing DNA fragmentation in NaF-treated GADD153^+/+^ and -^−/−^ MEF cells. GADD153^−/−^ cells had almost double the amount of DNA fragmentation compared with that observed in the GADD153^+/+^ cells, and this difference was attributable to caspase-3 activity (Fig. 6). GADD153 is generally considered a pro-apoptotic factor so we had expected that decreased GADD153 levels would reduce DNA fragmentation rather than increase it. Conversely, when GADD153 was overexpressed in LS8 cells, no difference in NaF-induced DNA fragmentation was observed. The downstream effects of GADD153 are not well characterized, and literature exists demonstrating that GADD153 affords protection from specific stressors. These stressors include H_2_O_2_ and O_2_ (59), radiation (60, 61), and human growth hormone, which induces apoptosis of mammary carcinoma cells (62). Recently it was demonstrated that GADD153 induces death by promoting protein synthesis and oxidation in the stressed ER (29). Thus, a reduced overall ER stress response that allows increased protein synthesis during critical ER protein overload will result in cell death. This sequence of events fits with our results. Because the UPR can lead to the activation of caspase-3 (18), a GADD153-mediated reduction in UPR induced caspase-3 activity could explain why DNA fragmentation was reduced in cells expressing GADD153. Therefore, by promoting protein synthesis, GADD153 may also have the downstream effect of reducing caspase-3 activity. Also, it should be noted that GADD153 is expressed during normal developmental processes such as adipogenesis (28), erythropoiesis, (63), and mammary gland development (64). Therefore, GADD153 plays other roles in addition to participating in apoptotic events.

To determine whether ER stress may play a role in dental fluorosis we adopted several approaches. Attempts to obtain enough ameloblast mRNA by use of laser-capture microdissection for real-time PCR analysis proved unsuccessful. The demineralization procedures necessary to section mouse incisors may have adversely affected the ameloblast mRNA. However, we did demonstrate that 150 ppm fluoride in drinking water virtually obliterated the mouse incisor ameloblast layer and resulted in a significant amount of apoptosis in the remaining cells (Fig. 7A). Previously it was demonstrated that mice will tolerate 175 ppm fluoride in their drinking water with no significant long-term (2 years) ill effects (35). Therefore, our result suggests that the ameloblasts are significantly more susceptible to the toxic effects of fluoride than are other tissues. In addition, immunohistochemical analysis of active Ire1 expression demonstrated that normal ameloblasts do express active Ire1 and that this expression may be enhanced by high dose fluoride treatment (Fig. 7, C and D, *right panel*). Although the mouse serum fluoride concentrations were significantly less than the concentrations necessary to induce ER stress in the LS8 cell line, the prolonged fluoride exposure (weeks) and a UPR that appears already active in endogenous ameloblasts may induce ER stress at substantially reduced fluoride concentrations. In any case, the ameloblasts are substantially more susceptible to the toxic effects of fluoride exposure than are other cells of the body, and we demonstrate here that ER stress may play a role in this susceptibility.

In summary, we have demonstrated that NaF induces an ER stress response in first passage EO cells and in the ameloblast-derived LS8 cell line. We also demonstrate that the ameloblasts of the enamel organ are, perhaps, the cells most susceptible to the adverse effects of high dose fluoride exposure. And, although further study is necessary, our data suggest that ER stress may play a role in dental fluorosis.

## Figures and Tables

**Table I:** Quantitative real-time PCR analysis of first passage porcine EO cells exposed to 2 mm NaF for 48 h Relative ratios represent the -fold induction of the specified mRNA relative to that of the internal control mRNA (eEF1*α*1). EO cells from two different pigs were assayed in duplicate.

Sample/replicate	GADD153 relative ratio	BiP relative ratio
EO Cells 1-A	29.7	4.1
EO Cells 1-B	17.3	4.7
EO Cells 2-A	107.3	10.0
EO Cells 2-B	330.3	6.8

**Table II:** Mouse serum fluoride concentrations Serum fluoride concentrations of mice treated for 3–4 weeks with 0, 75, or 150 ppm fluoride in drinking water.

Water fluoride	Serum fluoride			
	*μ*g/ml	*μ* m	Average	
			*μ*g/ml	*μ* m
0 ppm (0 *μ*m)	0.15	7.89	0.18 ± 0.017	9.47 ± 0.89
	0.18	9.47		
	0.17	8.95		
	0.19	10.00		
	0.19	10.00		
75 ppm (3947.37 *μ*m)	0.21	11.05	0.29 ± 0.069	15.26 ± 3.63
	0.26	13.68		
	0.35	18.42		
	0.35	18.42		
150 ppm (7894.74 *μ*m)	0.33	17.37	0.37 ± .081	19.47 ± 4.20
	0.31	16.32		
	0.36	18.95		
	0.49	25.79		

## Footnotes

1 The abbreviations used are: ER, endoplasmic reticulum; BiP, binding protein; CHOP, C/EBP homologous protein; CA-VI, carbonic anhydrase VI; GADD, growth arrest and DNA damage-inducible; MEF, mouse embryonic fibroblast; MTT, 3-(4,5-dimethylthiazol-2-yl)-2,5-diphenyltetrazolium bromide; UPR, unfolded protein response; Xbp-1, X-box-binding protein-1; ELISA, enzyme-linked immunosorbent assay; z-, benzyloxycarbonyl; siRNA, short interfering RNA; TUNEL, terminal dUTP nick-end labeling; EO, enamel organ; CMV, cytomegalovirus.

2 K. Lipson, L. Nguyen, S. Fonseca, E. Foss, R. Bortell, A. Rossini, and F. Urano, submitted for publication.

## References

[1] DenBesten PK (1999) Community Dent. Oral Epid 27, 41–47 10.1111/j.1600-0528.1999.tb01990.x 10086925

[2] Boivin G, Chavassieux P, Chapuy MC, Baud CA, and Meunier PJ (1989) Bone (NY) 10, 89–99 10.1016/8756-3282(89)90004-5 2765315

[3] Zager RA, and Iwata M (1997) Am. J. Pathol 150, 735–745 9033286 PMC1858266

[4] Thrane EV, Refsnes M, Thoresen GH, Lag M, and Schwarze PE (2001) Toxicol. Sci 61, 83–91 11294978 10.1093/toxsci/61.1.83

[5] Whitford GM (1996) in The Metabolism and Toxicity of Fluoride (Myers HM, ed) 2nd Ed., pp. 1–156, Karger, New York

[6] Evans RW, and Stamm JW (1991) J. Public Health Dent 51, 251–259 1941778 10.1111/j.1752-7325.1991.tb02223.x

[7] Evans RW, and Darvell BW (1995) J. Public Health Dent 55, 238–249 8551464 10.1111/j.1752-7325.1995.tb02376.x

[8] Smith CE (1998) Crit. Rev. Oral Biol. Med 9, 128–161 9603233 10.1177/10454411980090020101

[9] Smith CE, and Warshawsky H (1977) Anat. Rec 187, 63–98 835843 10.1002/ar.1091870106

[10] Bronckers AL, Lyaruu DM, Goei W, Litz M, Luo G, Karsenty G, Woltgens JH, and D’Souza RN (1996) Eur. J. Oral Sci 104, 102–111 8804897 10.1111/j.1600-0722.1996.tb00053.x

[11] Bronckers AL, Goei SW, Dumont E, Lyaruu DM, Woltgens JH, van Heerde WL, Reutelingsperger CP, and van den Eijnde SM (2000) Histochem. Cell Biol 113, 293–301 10857481 10.1007/s004180000137

[12] Kondo S, Tamura Y, Bawden JW, and Tanase S (2001) Arch. Oral Biol 46, 557–568 11311203 10.1016/s0003-9969(00)00139-4

[13] Nishikawa S, and Sasaki F (1995) Histochem. Cell Biol 104, 151–159 8536072 10.1007/BF01451574

[14] Schour I, and Smith M (1935) J. Am. Dent. Assoc 796–813

[15] Aoba T, and Fejerskov O (2002) Crit. Rev. Oral Biol. Med 13, 155–170 12097358 10.1177/154411130201300206

[16] Everett ET, McHenry MA, Reynolds N, Eggertsson H, Sullivan J, Kantmann C, Martinez-Mier EA, Warrick JM, and Stookey GK (2002) J. Dent. Res 81, 794–798 12407097 10.1177/0810794

[17] Vieira AP, Hancock R, Limeback H, Maia R, and Grynpas MD (2004) J. Dent. Res 83, 76–80 14691118 10.1177/154405910408300115

[18] Kaufman RJ (2002) J. Clin. Investig 110, 1389–1398 12438434 10.1172/JCI16886 PMC151822

[19] Harding HP, Calfon M, Urano F, Novoa I, and Ron D (2002) Annu. Rev. Cell Dev. Biol 18, 575–599 12142265 10.1146/annurev.cellbio.18.011402.160624

[20] Oyadomari S, and Mori M (2004) Cell Death Differ. 11, 381–389 14685163 10.1038/sj.cdd.4401373

[21] Liu CY, and Kaufman RJ (2003) J. Cell Sci 116, 1861–1862 12692187 10.1242/jcs.00408

[22] Calfon M, Zeng H, Urano F, Till JH, Hubbard SR, Harding HP, Clark SG, and Ron D (2002) Nature 415, 92–96 11780124 10.1038/415092a

[23] Yoshida H, Matsui T, Yamamoto A, Okada T, and Mori K (2001) Cell 107, 881–891 11779464 10.1016/s0092-8674(01)00611-0

[24] Wang Y, Shen J, Arenzana N, Tirasophon W, Kaufman RJ, and Prywes R (2000) J. Biol. Chem 275, 27013–27020 10856300 10.1074/jbc.M003322200

[25] Jin S, Antinore MJ, Lung FD, Dong X, Zhao H, Fan F, Colchagie AB, Blanck P, Roller PP, Fornace AJ Jr., and Zhan Q (2000) J. Biol. Chem 275, 16602–16608 10747892 10.1074/jbc.M000284200

[26] Harding HP, Zhang Y, Zeng H, Novoa I, Lu PD, Calfon M, Sadri N, Yun C, Popko B, Paules R, Stojdl DF, Bell JC, Hettmann T, Leiden JM, and Ron D (2003) Mol. Cell 11, 619–633 12667446 10.1016/s1097-2765(03)00105-9

[27] Jousse C, Bruhat A, Harding HP, Ferrara M, Ron D, and Fafournoux P (1999) FEBS Lett. 448, 211–216 10218478 10.1016/s0014-5793(99)00373-7

[28] Ron D, and Habener JF (1992) Genes Dev. 6, 439–453 1547942 10.1101/gad.6.3.439

[29] Marciniak SJ, Yun CY, Oyadomari S, Novoa I, Zhang Y, Jungreis R, Nagata K, Harding HP, and Ron D (2004) Genes Dev. 18, 3066–3077 15601821 10.1101/gad.1250704 PMC535917

[30] Chen LS, Couwenhoven RI, Hsu D, Luo W, and Snead ML (1992) Arch. Oral Biol 37, 771–778 1444889 10.1016/0003-9969(92)90110-t

[31] Bartlett JD, Luethy JD, Carlson SG, Sollott SJ, and Holbrook NJ (1992) J. Biol. Chem 267, 20465–20470 1400365

[32] Bertrand R, Kerrigan D, Sarang M, and Pommier Y (1991) Biochem. Pharmacol 42, 77–85 1648924 10.1016/0006-2952(91)90683-v

[33] Young CS, Kim SW, Qin C, Baba O, Butler WT, Taylor RR, Bartlett JD, Vacanti JP, and Yelick PC (2005) Arch. Oral Biol 50, 259–265 15721159 10.1016/j.archoralbio.2004.11.020

[34] Pfaffl MW (2001) Nucleic Acids Res. 29, 2002–2007 10.1093/nar/29.9.e45 PMC55695 11328886

[35] National Toxicology Program (1990) Natl. Toxicol. Program Tech. Rep. Ser 393, 1–448 12637966

[36] Rojas-Sanchez F, Kelly SA, Drake KM, Eckert GJ, Stookey GK, and Dunipace AJ (1999) Community Dent. Oral Epid 27, 288–297 10.1111/j.1600-0528.1998.tb02023.x 10403089

[37] Vogel GL, Carey CM, Chow LC, and Brown WE (1987) J. Dent. Res 66, 1691–1697 10872409 10.1177/00220345870660111801

[38] Kaufman RJ (1999) Genes Dev. 13, 1211–1233 10346810 10.1101/gad.13.10.1211

[39] Price BD, and Calderwood SK (1992) Cancer Res. 52, 3814–3817 1617653

[40] Wang XZ, Kuroda M, Sok J, Batchvarova N, Kimmel R, Chung P, Zinszner H, and Ron D (1998) EMBO J. 17, 3619–3630 9649432 10.1093/emboj/17.13.3619 PMC1170698

[41] Sok J, Wang XZ, Batchvarova N, Kuroda M, Harding H, and Ron D (1999) Mol. Cell. Biol 19, 495–504 9858573 10.1128/mcb.19.1.495 PMC83907

[42] Anuradha CD, Kanno S, and Hirano S (2000) Arch. Toxicol 74, 226–230 10959797 10.1007/s002040000132

[43] Ron D (2002) J. Clin. Investig 110, 1383–1388 12438433 10.1172/JCI16784 PMC151821

[44] Holland RI (1979) Acta Pharmacol. Toxicol 45, 96–101 10.1111/j.1600-0773.1979.tb02367.x 40394

[45] Zhou R, Zaki AE, and Eisenmann DR (1996) Arch. Oral Biol 41, 739–747 9022911 10.1016/s0003-9969(96)00078-7

[46] Matsuo S, Inai T, Kurisu K, Kiyomiya K, and Kurebe M (1996) Arch. Toxicol 70, 420–429 8740536 10.1007/s002040050294

[47] Smith CE, Nanci A, and DenBesten PK (1993) Anat. Rec 237, 243–258 8238976 10.1002/ar.1092370212

[48] Elliott J, Scarpello JH, and Morgan NG (2002) J. Endocrinol 172, 137–143 11786381 10.1677/joe.0.1720137

[49] Hirano S, and Ando M (1996) Arch. Toxicol 70, 249–251 8825685 10.1007/s002040050268

[50] Refsnes M, Kersten H, Schwarze PE, and Lag M (2002) Ann. N. Y. Acad. Sci 973, 218–220 12485864 10.1111/j.1749-6632.2002.tb04636.x

[51] Refsnes M, Schwarze PE, Holme JA, and Lag M (2003) Hum. Exp. Toxicol 22, 111–123 12723891 10.1191/0960327103ht322oa

[52] Hirano S, and Ando M (1997) Arch. Toxicol 72, 52–58 9458191 10.1007/s002040050468

[53] Zhang J, Liu X, Scherer DC, van Kaer L, Wang X, and Xu M (1998) Proc. Natl. Acad. Sci. U. S. A 95, 12480–12485 9770511 10.1073/pnas.95.21.12480 PMC22856

[54] Chang HY, and Yang X (2000) Microbiol. Mol. Biol. Rev 64, 821–846 11104820 10.1128/mmbr.64.4.821-846.2000 PMC99015

[55] Sterling D, Reithmeier RA, and Casey JR (2001) J. Pancreas 2, 165–170 11875254

[56] Simmer JP, and Fincham AG (1995) Crit. Rev. Oral Biol. Med 6, 84–108 7548623 10.1177/10454411950060020701

[57] Smith CE, Chong DL, Bartlett JD, and Margolis HC (2005) J. Bone Miner. Res 20, 240–249 15647818 10.1359/JBMR.041002

[58] Sui W, Boyd C, and Wright JT (2003) J. Dent. Res 82, 388–392 12709507 10.1177/154405910308200512

[59] Guyton KZ, Spitz DR, and Holbrook NJ (1996) Free Radic. Biol. Med 20, 735–741 8721617 10.1016/0891-5849(95)02151-5

[60] Chen C, Nussenzweig A, Guo M, Kim D, Li GC, and Ling CC (1996) Oncogene 13, 1659–1665 8895511

[61] Mayerhofer T, and Kodym R (2003) Biochem. Biophys. Res. Commun 310, 115–120 14511657 10.1016/j.bbrc.2003.08.130

[62] Mertani HC, Zhu T, Goh EL, Lee KO, Morel G, and Lobie PE (2001) J. Biol. Chem 276, 21464–21475 11297545 10.1074/jbc.M100437200

[63] Coutts M, Cui K, Davis KL, Keutzer JC, and Sytkowski AJ (1999) Blood 93, 3369–3378 10233889

[64] Talukder AH, Wang RA, and Kumar R (2002) Oncogene 21, 4289–4300 12082616 10.1038/sj.onc.1205529

